# Supplementary figures and images for: First ex situ outplanting of the habitat-forming seaweed Cystoseira amentacea var. stricta from a restoration perspective
Source: PeerJ. 2019 Jul 22;7:e7290. doi: 10.7717/peerj.7290 (PMC6657741; doi:10.7717/peerj.7290)

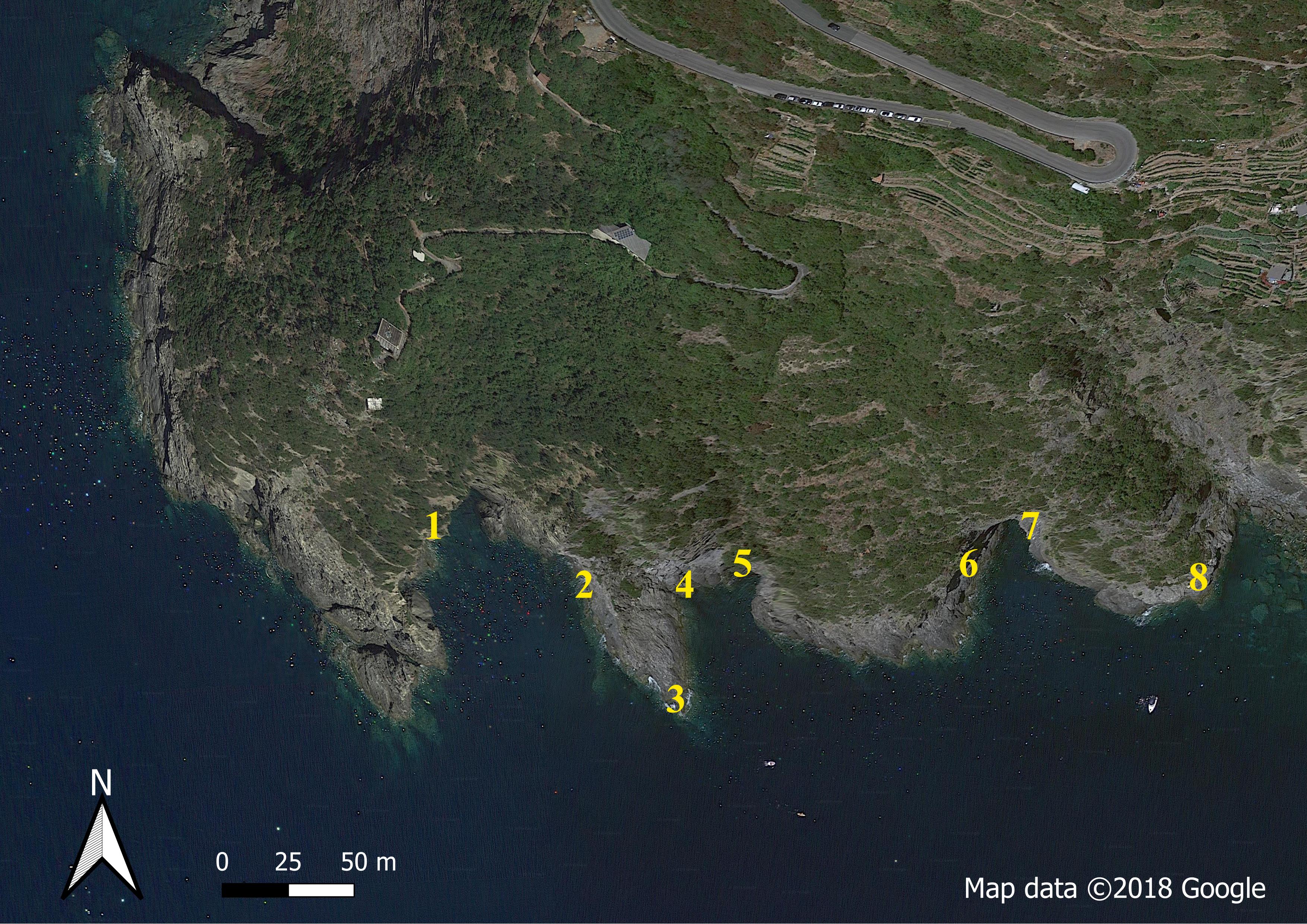

Supplement: Supplemental Information 1 — Map data ©2018 Google using QGIS v 3.4.4. [file peerj-07-7290-s001.jpg]
